# YAP mechanotransduction under cyclic mechanical stretch loading for mesenchymal stem cell osteogenesis is regulated by ROCK

**DOI:** 10.3389/fbioe.2023.1306002

**Published:** 2024-01-11

**Authors:** Eunju Kim, Brandon D. Riehl, Tasneem Bouzid, Ruiguo Yang, Bin Duan, Henry J. Donahue, Jung Yul Lim

**Affiliations:** ^1^ Department of Mechanical and Materials Engineering, College of Engineering, University of Nebraska-Lincoln, Lincoln, NE, United States; ^2^ Department of Internal Medicine, College of Medicine, University of Nebraska Medical Center, Omaha, NE, United States; ^3^ Department of Biomedical Engineering, College of Engineering, Virginia Commonwealth University, Richmond, VA, United States

**Keywords:** mechanical stretch loading, mesenchymal stem cell, osteogenesis, YAP, Hippo, ROCK

## Abstract

While yes-associated protein (YAP) is now recognized as a potent mechanosensitive transcriptional regulator to affect cell growth and differentiation including the osteogenic transcription of mesenchymal stem cells (MSCs), most studies have reported the YAP mechanosensing of static mechanophysical cues such as substrate stiffness. We tested MSC response to dynamic loading, i.e., cyclic mechanical stretching, and assessed YAP mechanosensing and resultant MSC osteogenesis. We showed that cyclic stretching at 10% strain and 1 Hz frequency triggered YAP nuclear import in MSCs. YAP phosphorylation at S127 and S397, which is required for YAP cytoplasmic retention, was suppressed by cyclic stretch. We also observed that anti-YAP-regulatory Hippo pathway, LATS phosphorylation, was significantly decreased by stretch. We confirmed the stretch induction of MSC osteogenic transcription and differentiation, and this was impaired under YAP siRNA suggesting a key role of YAP dynamic mechanosensing in MSC osteogenesis. As an underlying mechanism, we showed that the YAP nuclear transport by cyclic stretch was abrogated by ROCK inhibitor, Y27632. ROCK inhibitor also impaired the stretch induction of F-actin formation and MSC osteogenesis, thus implicating the role of the ROCK-F-actin cascade in stretch-YAP dynamic mechanosensing-MSC osteogenesis. Our results provide insight into bone tissue engineering and skeletal regenerative capacity of MSCs especially as regards the role of dynamic mechanical loading control of YAP-mediated MSC osteogenic transcription.

## 1 Introduction

Cells sense and respond to extracellular mechanical milieus, both static and dynamic, through the process called mechanotransduction (Bouzid et al., 2019). In such a cellular adaptation process, signals from static extracellular mechanical environments (e.g., matrix stiffness, topography, mechanical condition provided by micropatterned cell size, etc.,) and dynamic external mechanical loading cues (e.g., mechanical stretch, fluid shear, compression, etc.,) are transduced to cells to regulate intracellular signaling cascades. This ultimately induces changes in transcription, gene expression, protein synthesis, and downstream molecular signaling. Mechanotransduction plays a crucial role in cellular growth, homeostasis, and differentiation, and its breakdown is implicated in various pathological conditions including osteoporosis, muscular dystrophy, and even cardiac failure and cancer (Panciera et al., 2017; Li et al., 2022).

Mesenchymal stem cells (MSCs) are a specialized population of progenitor cells that can differentiate into various lineages, including osteogenic, adipogenic, and myogenic. The differentiation of MSCs depending on extracellular milieus plays a pivotal role in multiple cell physiology. Especially, the balance of MSC osteogenic vs. adipogenic commitment and differentiation within the bone marrow environment is vital for healthy bone homeostasis (Chen et al., 2016). Traditionally, biochemical cues have been examined as to their ability to affect osteogenic or adipogenic MSC lineage commitment. For example, exposure to varying types of bone morphogenetic proteins (BMPs) results in MSC osteogenesis or adipogenesis (Noël et al., 2004; Tang et al., 2004). It has been also established that mechanophysical cues, such as mechanical stretch loading, can direct MSC osteogenesis or adipogenesis (Lee et al., 2012; Song et al., 2018). For both biochemical and mechanophysical cues, underlying molecular mechanisms of MSC fate decision to osteoblast or adipocyte are, however, not fully revealed. See the review by Takada et al. (2009) on relatively well-established Wnt and PPARγ signaling.

Recently, studies focusing on MSC osteogenesis suggest that yes-associated protein (YAP), with its homolog transcriptional co-activator with PDZ-binding motif (TAZ), plays a central role in initiating osteogenic transcription in MSCs (Piccolo et al., 2014; Wei et al., 2020). In many cell types, YAP/TAZ, as a potential downstream effector of the Hippo pathway, acts to govern cell proliferation and differentiation through nuclear accumulation and consequent transcriptional activation (Feng et al., 2016; Huang et al., 2016). When the Hippo pathway is upregulated, YAP becomes phosphorylated and thus inactivated, leading to YAP cytoplasmic retention via binding with protein 14-3-3 resulting in YAP protein degradation within the cytoplasm. When dephosphorylated, YAP can translocate into the nucleus and then associate with the TEAD family of transcription factors to stimulate their actions, including RUNX2 osteogenic transcription. It has been reported that such cytoplasmic vs. nuclear localization of YAP and respective transcriptional deactivation vs. activation can be controlled by extracellular mechanophysical cues. For example, cell seeding on a rigid substrate induces nuclear translocation of YAP to promote osteogenic differentiation of MSCs, but this does not occur on a soft substrate (Chen et al., 2021a). In addition, MSCs micropatterned to spread into a large cell spreading area display YAP nuclear localization and osteogenic transcription, relative to those patterned into a small area (Jiao et al., 2020).

It is noteworthy that the vast majority of studies on the mechanophysical control of YAP for MSC osteogenesis has dealt with static extracellular mechanophysical cues—matrix stiffness and cell micropatterning size, as referenced above. In contrast, very little is known in respect to dynamic mechanical loading control of YAP mechanotransduction for MSC osteogenic transcription. This significantly hinders the advancements of both fundamental understanding and practical application of functional bone tissue engineering and skeletal regenerative medicine that commonly deal with mechanical loading cues, such as mechanical stretching, to achieve enhanced bone formation with MSCs. A few recent studies reported mechanical stretch loading induction of osteogenic differentiation via YAP signaling in periodontal ligament cells (Yang et al., 2018) and fibroblasts (Peng et al., 2021). Another recent study reported that mechanically stretched macrophages displayed upregulated YAP activity, and co-culture of MSCs with stretched macrophages showed increased osteogenesis (Dong et al., 2021). Intriguingly, two recent studies reported contrasting results on YAP nuclear transport in MSCs under mechanical stretch loading. Robert Mauck’s group showed that YAP nuclear transport was activated by cyclic stretching at 3% strain and 1 Hz frequency (Driscoll et al., 2015). On the other hand, Janet Rubin’s group reported that cyclic stretching at 2% strain and 10 cycles/min did not induce YAP nuclear import (Sen et al., 2022). However, these two studies, both of which examined bone marrow MSCs, did not test the impact of YAP on MSC osteogenic transcription and differentiation.

Here, we report the MSC response to cyclic mechanical stretch loading in YAP activation and resultant osteogenic transcription and terminal differentiation. We showed that in MSCs YAP is translocated to the nucleus following cyclic stretch loading with decreased YAP phosphorylation. We also observed that anti-YAP Hippo signaling was impaired by cyclic stretch. Further, we revealed the potential mechanism that RhoA kinase (ROCK) regulates stressed actin filament (F-actin) formation under stretch, which in turn governs the cyclic stretch triggering of YAP nuclear import and consequent induction of MSC osteogenic transcription. This is, to our best knowledge, the first study that reports mechanical stretch loading control of Hippo and YAP mechanosignaling in MSCs and the regulatory role of ROCK and F-actin in the process of directing MSC osteogenic fate decision via YAP.

## 2 Materials and methods

### 2.1 Cell culture

Murine MSCs (C3H10T1/2, ATCC, CCL-226) were seeded using growth medium composed of Dulbecco’s modified Eagle’s medium (DMEM) with 10% fetal bovine serum (FBS) and 1% penicillin/streptomycin (P/S). For inducing osteogenesis, growth medium was replaced by osteogenic differentiation medium (further containing 10 mM β-glycerophosphate, 10 nM dexamethasone, and 50 μg/mL ascorbic acid) when cells reached confluence. The medium was changed every 2-3 days. MSC osteogenesis was evaluated by qRT-PCR and western blotting of osteogenic transcription factor and genes on day 7 (after giving osteogenic media) and Alizarin red staining of synthesized bone minerals on day 21. The key experiment of YAP nuclear translocalization under stretch loading was repeated with human mesenchymal stem cells (hMSCs, Lonza, PT-2501, 25 year-old female) using mesenchymal stem cell growth medium (Lonza, PT-3001). All experiments throughout this study were done on type-I collagen-coated cell stretch plates (Flexcell International, BioFlex).

### 2.2 Mechanical stretch loading of cells

Cyclic mechanical stretching of cells was conducted using the FX-5000 Tension device (Flexcell International) as we previously described (Lee et al., 2012; Lee et al., 2015; Shradhanjali et al., 2017). Briefly, cells seeded on the BioFlex stretch plate were subjected to cyclic tensile stretching at 10% strain and 1 Hz frequency. In the device, pulling-down of the cell-seeded elastic membrane against a circular loading post provides equiaxial membrane elongation at well-defined stretch regimens. For imaging tests, cells were seeded in the 6-well BioFlex stretch plate at 3 × 10^5^ cells per each well. For immunoblotting and osteogenesis tests, cells were seeded at 5 × 10^5^ cells per well. For the YAP nuclear import test, cells were seeded for 24 h and then stretched. For osteogenesis tests, stretching was applied for 3 days (1 h per day) with the onset of the osteogenic induction. The control (unstretched) cells were incubated under the same experimental condition in the same stretch plate but not subjected to stretch loading. The stretch plates were housed inside the incubator, thereby all tests of cell growing and stretch loading, YAP nuclear import, and osteogenesis were completed at 37°C in a humidified atmosphere with 5% CO_2_.

### 2.3 Immunocytochemistry

Cells were fixed with 4% paraformaldehyde solution for 20 min and permeabilized with 0.1% Triton X-100 for 5 min. After blocking for 30 min with 3% bovine serum albumin (BSA), cells were incubated overnight at 4°C with anti-YAP antibody (1:100, Santa Cruz, SC-101199). Cells were washed with phosphate-buffered saline (PBS) three times, and incubated with Alexa Fluor 488-conjugated secondary antibody (1:500, Jackson Immunoresearch) for 1 h. DAPI (1:1,000, Santa Cruz, SC-3598) and rhodamine phalloidin (1:1,000, Invitrogen, R415) were used to stain nucleus and F-actin, respectively. Fluorescent images were taken with a Zeiss LSM 800 confocal microscope, and nuclear localization of YAP was analyzed using a NIH ImageJ software (see figure captions for assessing cells positive for YAP nuclear localization).

### 2.4 Western immunoblotting

Either whole cell lysates, or separated nuclear and cytoplasmic fractions were used for western blot analyses. Cells were lysed with radioimmunoprecipitation (RIPA) buffer (Thermo Fisher, PI89900) with a protease-inhibitor cocktail (Sigma Aldrich, S8830) and phosphatase inhibitor (Sigma Aldrich, P2850). Whole cell lysates were incubated on ice for 30 min and then centrifuged at 14,000 g for 20 min at 4°C. The supernatants were collected and used for western blotting. For the nuclear and cytoplasmic fractionation, a Nuclear and Cytoplasmic Extraction Reagents Kit (Thermo Fisher, PI-78835) was used following the manufacturer’s protocol. For western immunoblotting, proteins were loaded on sodium dodecyl sulfate-polyacrylamide gel electrophoresis (SDS-PAGE) gels and transferred to polyvinylidene fluoride (PVDF) membranes. After blocking with 5% skim milk, the membranes were incubated overnight at 4°C with primary antibodies: anti-YAP (1:1,000, Santa Cruz, SC-101199), anti-phosphorylated YAP (p-YAP) at Ser127 (1:1,000, Cell Signaling, 4911S), anti-p-YAP at Ser397 (1:1,000, Cell Signaling, 13619S), anti-LATS1 (1:500, Cell Signaling, 3477T), anti-phosphorylated LATS1 (p-LATS1) (1:1,000, Cell Signaling, 8654S), anti-RUNX2 (1:1,000, Cell Signaling, 125567S), anti-GAPDH (1:2,000, Santa Cruz, SC-32233), and anti-lamin B1 (1:1,000, Abcam, ab184115). After washing three times, immunoblots were probed with the horseradish peroxidase (HRP)-linked anti-mouse or anti-rabbit IgG secondary antibodies (1:3,000, Jackson Immunoresearch) for 40 min. Protein immunoreactivity was detected by electrogenerated chemiluminescence (ECL), and immunoblotting band intensities were obtained by NIH ImageJ. Finally, the relative expression strength of the target protein band was quantified by normalizing to the band strength of the loading control, GAPDH or lamin B1.

### 2.5 siRNA and inhibitor

For knockdown tests, small interfering RNAs (siRNAs) purchased from Santa Cruz Biotechnology were used: YAP siRNA (SC-38638) and control siRNA (SC-37007). Transfection of siRNA was performed using a Lipofectamine RNAiMAX (Invitrogen, 13778075) following the manufacturer’s protocol. Briefly, 4 µL siRNA and 4 µL RNAiMAX were diluted in a reduced serum medium (Gibco, 31985070), and cells were transfected with the siRNA mixture. The effectiveness of siRNA silencing was confirmed using western blotting. For the inhibitor assay, cells were treated with 10 µM of Y27632 (ROCK inhibitor, Selleck Chemical, S1049) in dimethyl sulfoxide (DMSO) vehicle. For no Y27632 groups, we used the same amount of DMSO as control vehicle samples.

### 2.6 Real-time quantitative PCR

The total RNA extraction was done using a TRIzol reagent (Invitrogen). The concentration of RNA was quantified by utilizing a NanoDrop spectrophotometer. Complementary DNA (cDNA) was synthesized using a Reverse Transcription Supermix Kit (Bio-Rad, 1708841) according to the manufacturer’s protocol. The qRT-PCR reaction was carried out on a Mastercycler RealPlex2 real-time PCR system (Eppendorf) using the Power-up SYBR Green Master Mix (Applied Biosystems, A25741) with gene-specific primers. Relative mRNA expression was calculated by normalizing to GAPDH mRNA levels.

### 2.7 Alizarin red staining

Bone-like mineral formation was evaluated by staining with 2% Alizarin red solution (Sigma Aldrich, A5533). Cells were washed with PBS and fixed with 10% formalin. After washing, cells were incubated with Alizarin red solution for 45 min in the dark. In addition to imaging of the stained cells, calcium content was quantified. For this, calcium extracts by Alizarin red staining were obtained by 10% cetylpyridinium chloride solution (Sigma Aldrich, C0732), and relative calcium content was quantified by optical absorbance at 405 nm in a spectrophotometer (BioTek).

### 2.8 Statistics

All experiments were conducted at least as three independent tests, and the results were presented in the figures as average ± standard error of measurements. Statistical significance between test groups was assessed by one-way analysis of variance (ANOVA) followed by Fisher’s Least Significant Difference (LSD) post-hoc test, and levels of significance were marked as *: *p* < 0.05, **: *p* < 0.01, and ***: *p* < 0.001. The statistical analysis was performed in MATLAB version 9.13 (R2022b) using the Statistics and Machine Learning Toolbox version 12.4 (R2022b).

## 3 Results

### 3.1 Cyclic mechanical stretch loading promotes MSC osteogenic differentiation

Before testing YAP mechanotransduction in MSCs under cyclic mechanical stretch loading, we confirmed stretch induction of MSC osteogenic differentiation. When murine C3H10T1/2 MSCs were cyclically stretched, MSCs exhibited increases in osteogenic transcription factor and gene expression, including RUNX2, alkaline phosphatase (ALP), type-1 collagen (COL-I), and osteopontin (OPN), compared with control (osteogenic media but without stretch) (Supplementary Figure S1). Terminal bone-like mineralization was also greater for the stretched group. These results indicate that cyclic stretch loading promotes MSC osteogenesis. Additionally, we tested the effect of cyclic stretch on MSC adipogenesis, considering that mechanical load-induced MSC osteogenesis comes at the expense of adipogenesis (David et al., 2007). Cyclic stretch applied during the adipogenic induction suppressed lipid accumulation and adipogenic marker expressions (PPARγ and FAS) in MSCs (Supplementary Figure S2).

### 3.2 Cyclic mechanical stretch loading induces YAP nuclear translocalization in MSCs

**FIGURE 1:**
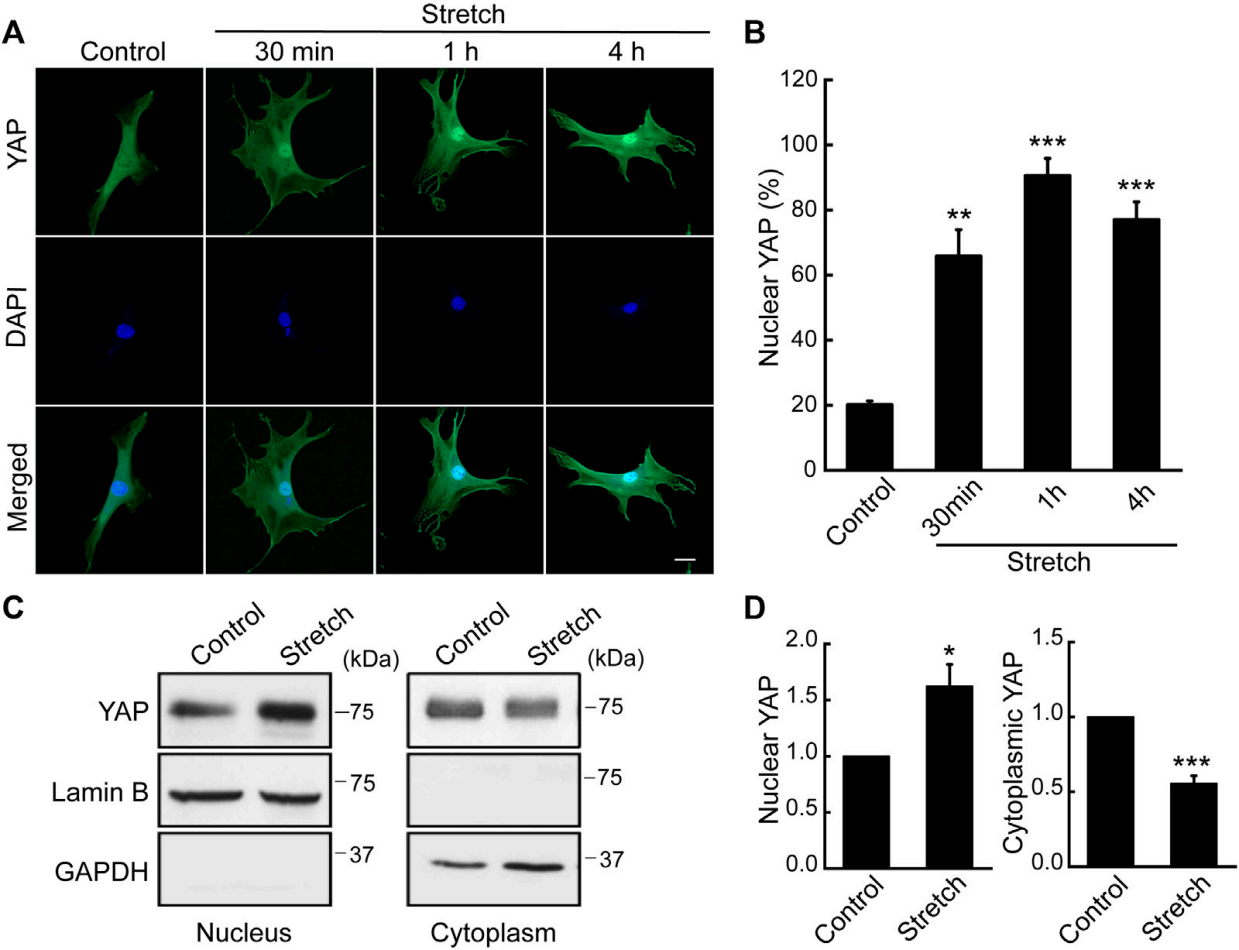
Cyclic stretch loading induces YAP nuclear translocalization in MSCs. C3H10T1/2 murine MSCs were unstretched (control) or stretched, and assessed for immunostaining and western blotting for YAP. **(A,B)** Immunofluorescence of YAP (green) and DAPI (blue). Assessment of cells showing nuclear YAP localization was conducted using the NIH ImageJ analysis of YAP immunofluorescence. For this, the ratio of nuclear/cytoplasmic YAP fluorescent intensity was quantified for each cells. Then, the average and standard deviation of the YAP intensity ratios of the unstretched control cells were obtained. In case a cell showed a YAP nuclear/cytoplasmic fluorescent intensity ratio greater than the average plus one standard deviation of the control, then the cell was determined as to exhibit YAP nuclear residency. Finally, the percentage of cells with YAP nuclear localization was obtained (n of 30 or more cells per each condition). Scale bar = 20 μm. **(C,D)** Western blot analysis of nuclear and cytoplasmic protein extracts from MSCs stretched for 1 h. Anti-lamin B1 was used as a loading control for the nuclear fraction and anti-GAPDH for the cytosolic fraction (*n* = 3). *: *p* < 0.05, **: *p* < 0.01, and ***: *p* < 0.001 compared with control.

As a potential regulatory mechanism for stretch induction of MSC osteogenesis, we investigated YAP nuclear transport induced by stretch. While subcellular location of YAP was seen throughout the cytoplasm and nucleus in unstretched MSCs, cyclic stretch triggered YAP translocation to the nucleus (Figure 1A). This was quantified by the percentage of cells positive for YAP nuclear localization using immunocytochemistry and NIH ImageJ analysis (Figure 1B). We observed that YAP nuclear localization in MSCs was the greatest at 1 h cyclic stretching under 10% strain and 1 Hz frequency, and used these regimens in subsequent tests on YAP and osteogenesis. YAP nuclear import by stretch was further evaluated by using nuclear and cytoplasmic protein fractionation and immunoblotting with appropriate loading controls (Figures 1C, D). It was confirmed that cyclic stretch loading significantly increases YAP protein level inside the nucleus while decreasing YAP cytoplasmic expression. For additional validation with human MSCs (Lonza), we repeated the stretch test using the same stretch regimens and observed an analogous trend (Supplementary Figure S3). This implies a potential universal trend in YAP dynamic mechanosensing in MSCs in response to external mechanical stretch loading.

### 3.3 Cyclic stretch suppresses YAP phosphorylation and anti-YAP-regulatory Hippo pathway, LATS phosphorylation

**FIGURE 2:**
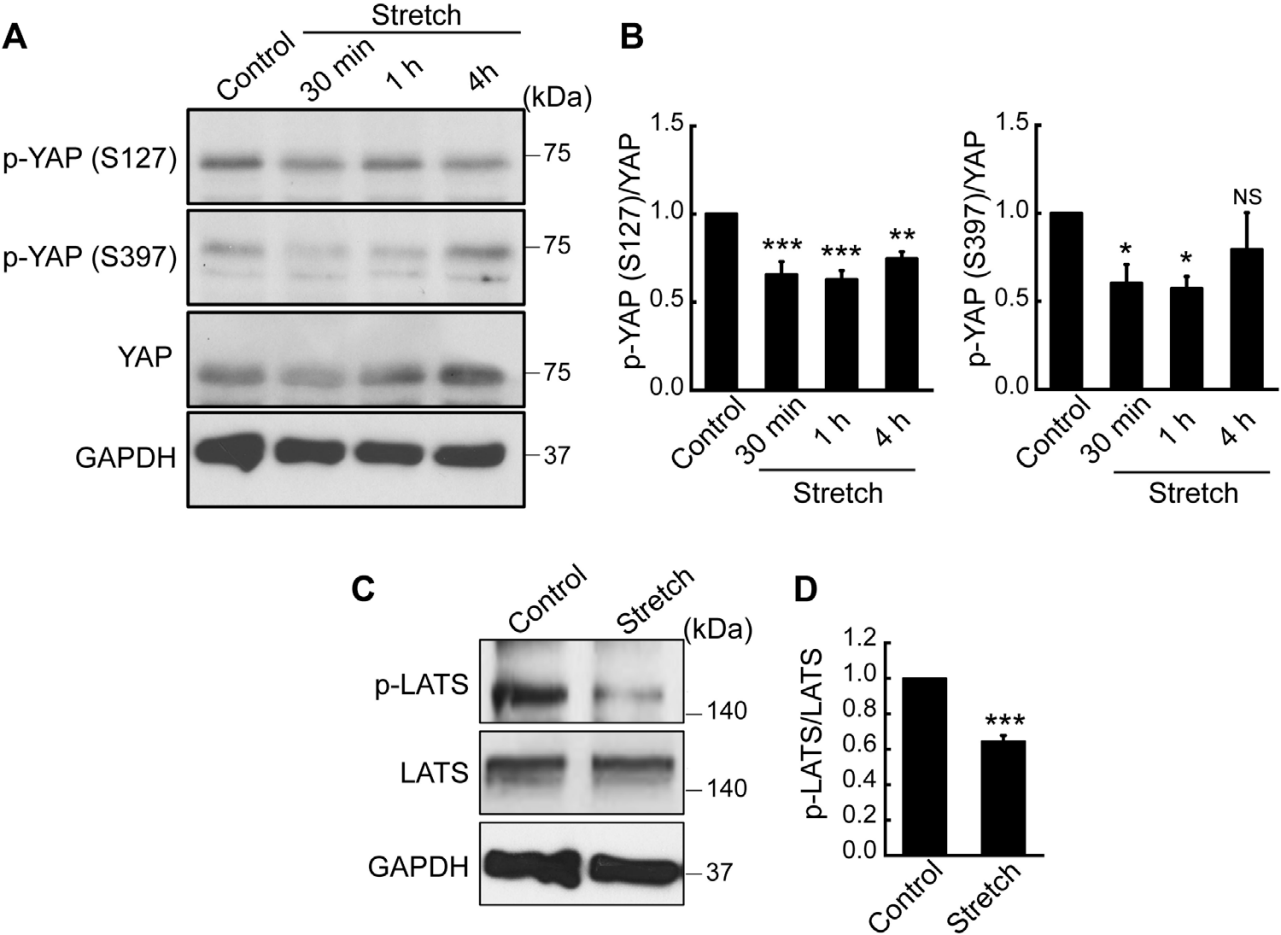
Cyclic stretch loading suppresses YAP phosphorylation and downregulates the key Hippo pathway, LATS phosphorylation. **(A,B)** MSCs were stretched and YAP phosphorylation was assessed by western blotting. The expression levels of p-YAP (S127) and p-YAP (S397) were decreased by stretch, indicating that YAP cytoplasmic retention via phosphorylation was suppressed by stretch. The ratio of p-YAP/total YAP was quantified (*n* = 4). **(C,D)** The phosphorylation of LATS is a key Hippo pathway that acts as a potential anti-YAP upstream effector. LATS phosphorylation was impaired under cyclic stretch loading for 30 min. The ratio of p-LATS1/total LATS1 was quantified (*n* = 3). NS: non-significant, *: *p* < 0.05, **: *p* < 0.01, and ***: *p* < 0.001 compared with control.

Hippo signaling is a potential upstream pathway of YAP. If Hippo signaling is upregulated via the phosphorylation of LATS, YAP can become phosphorylated, thereby resulting in YAP cytoplasmic retention and degradation. On the contrary, dephosphorylated YAP is able to translocate to the nucleus for its activation. To test the effect of mechanical stretch loading on these mechanisms, MSCs were stretched and YAP phosphorylation was assessed. YAP has five phosphorylation sites (S61, S109, S127, S164, and S397), among which the phosphorylation at S127 and S397 by the Hippo effector, LATS, is known to be responsible for YAP cytoplasmic retention and degradation (Moon et al., 2017). When cyclically stretched, MSCs displayed marked decreases in endogenous YAP phosphorylation at both S127 and S397 sites (Figures 2A, B). The time course showed significant decreases at these sites up to 1 h stretching. Furthermore, phosphorylation of LATS1 (p-LATS1) was significantly suppressed by 30 min of cyclic stretching (Figures 2C, D), thereby proposing that Hippo pathway such as LATS could be involved in the stretch regulation of YAP. Collectively, our results indicate that cyclic stretch loading facilitates YAP nuclear transport in MSCs via downregulating YAP phosphorylation and its cytoplasmic retention and that stretch regulation of anti-YAP Hippo pathway, p-LATS, may play an associated role.

### 3.4 Knockdown of YAP by siRNA inhibits cyclic stretch induction of MSC osteogenesis

**FIGURE 3:**
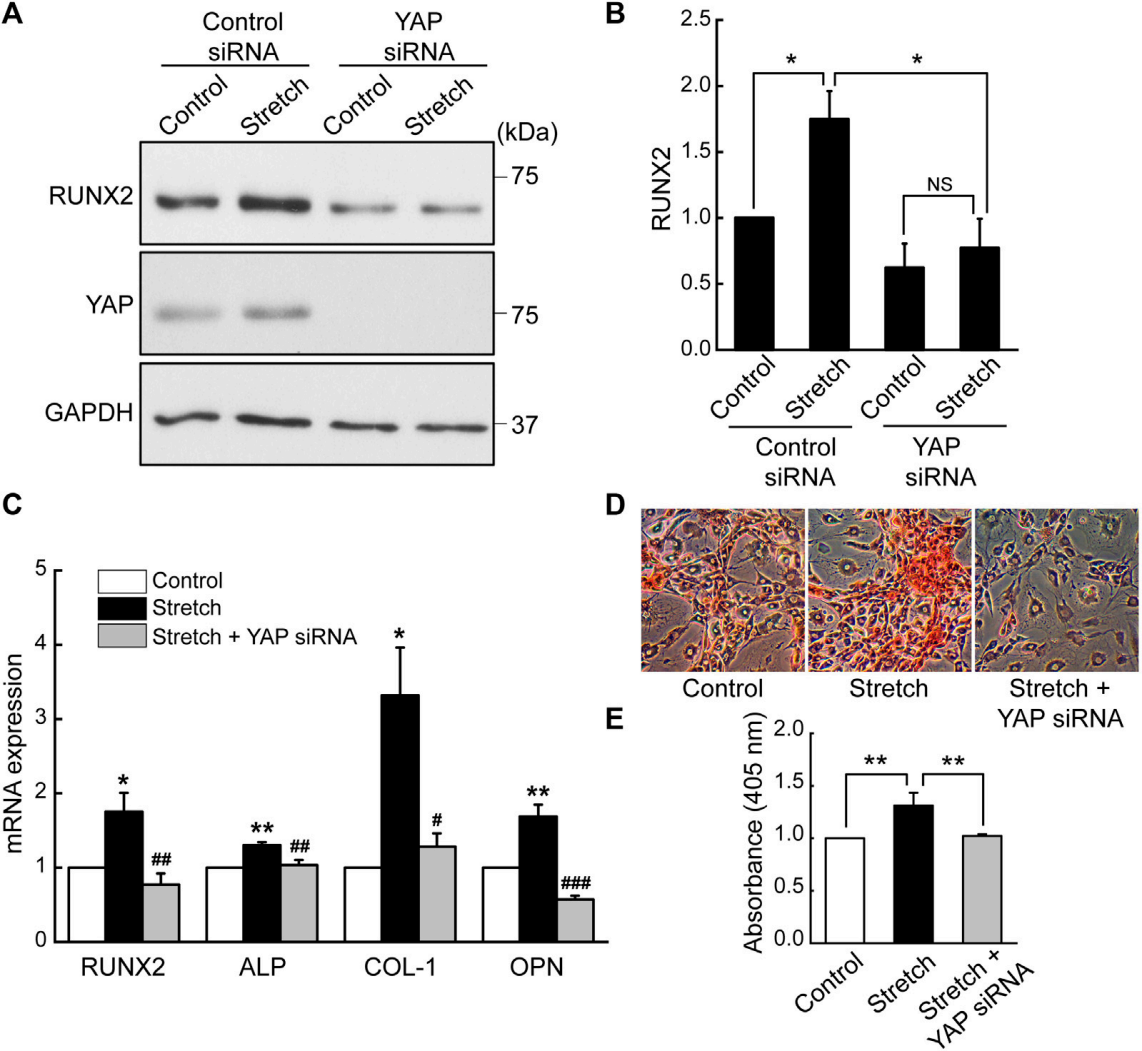
Cyclic stretch induction of MSC osteogenic transcription and differentiation is impaired by YAP silencing. **(A,B)** MSCs were stretched without or with YAP siRNA, and the key osteogenic transcription factor, RUNX2, was assessed by western blotting on day 7 (*n* = 4). YAP siRNA was given with the start of the osteogenic induction and stretch. **(C)** Osteogenic gene expressions by qRT-PCR on day 7. **(D,E)** Alizarin red bone mineral staining on day 21 (*n* = 3). For **(C–E)**, control (no stretch) and stretch without YAP siRNA was tested under control siRNA. NS: non-significant; *: *p* < 0.05 and **: *p* < 0.01 compared with control or corresponding counterpart; ^#^: *p* < 0.05, ^##^: *p* < 0.01, and ^###^: *p* < 0.001 for comparison between stretch vs. stretch + YAP siRNA.

Based on the finding that cyclic stretch loading induces nuclear transport of a key transcriptional regulator, YAP, we examined the role of YAP in the stretch induction of MSC osteogenesis by transfecting MSCs with YAP siRNA. The efficiency of YAP siRNA knockdown was tested by western blotting (Supplementary Figure S4). It was observed that the stimulatory effect of cyclic stretching on the protein expression of a key osteogenic transcription factor, RUNX2, was abrogated in the presence of YAP siRNA (Figures 3A, B). Also, YAP silencing abolished stretch-induced increases in osteogenic transcription factor and gene markers, RUNX2, ALP, COL-I, and OPN, as assessed by qRT-PCR (Figure 3C). Consistent with these data, MSCs displayed enhanced bone-like mineralization by stretch and this effect was impaired by YAP siRNA (Figures 3D, E). These results demonstrate that the promotion of MSC osteogenic transcription and differentiation by cyclic stretch is dependent on the functional action of YAP.

### 3.5 ROCK regulates cyclic stretch-induced YAP nuclear transport and F-actin formation

**FIGURE 4:**
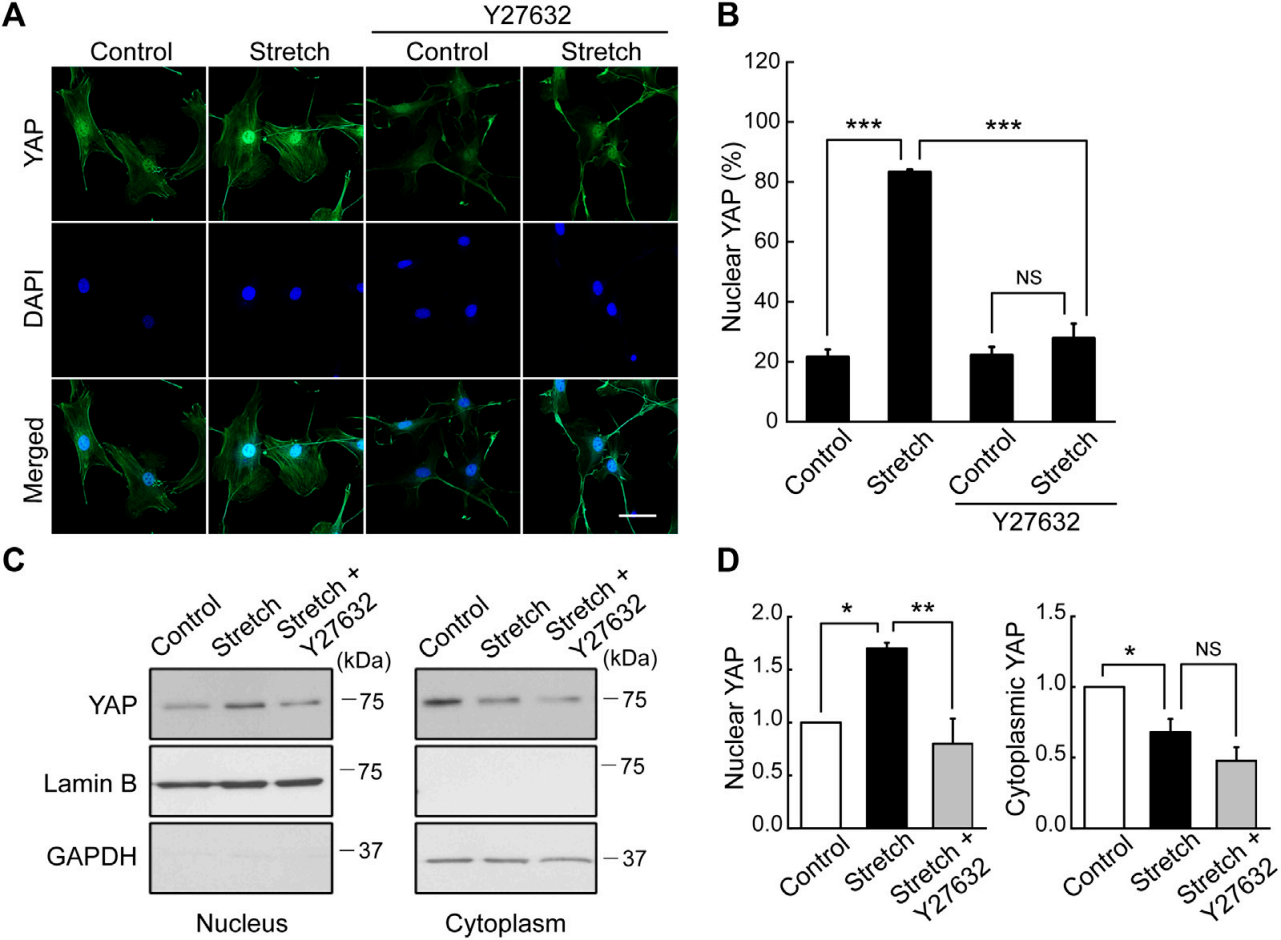
YAP nuclear transport by stretch is abrogated by ROCK inhibitor. **(A,B)** MSCs were stretched under a ROCK inhibitor (Y27632) for 1 h, and YAP nuclear localization was assessed by immunostaining of YAP (green) and DAPI (blue). The stretch induction of YAP nuclear import was suppressed by Y27632. This was assessed with the percentage cells positive for YAP nuclear localization, as quantified from the nuclear/cytoplasmic YAP fluorescent intensity ratios (n of 36 cells or more per each condition) using the methods described in Figure 1B. Scale bar = 20 μm. **(C,D)** YAP expression was assessed by western blotting for nuclear and cytoplasmic extracts with appropriated loading controls, lamin B1 for the nuclear fraction and GAPDH for the cytosolic fraction (*n* = 3). Throughout the experiment, the cases without Y27632 were tested under the same amount of DMSO used in Y27632 inhibitor cases as a delivery vehicle. NS: non-significant, *: *p* < 0.05, **: *p* < 0.01, ***: *p* < 0.001.

**FIGURE 5:**
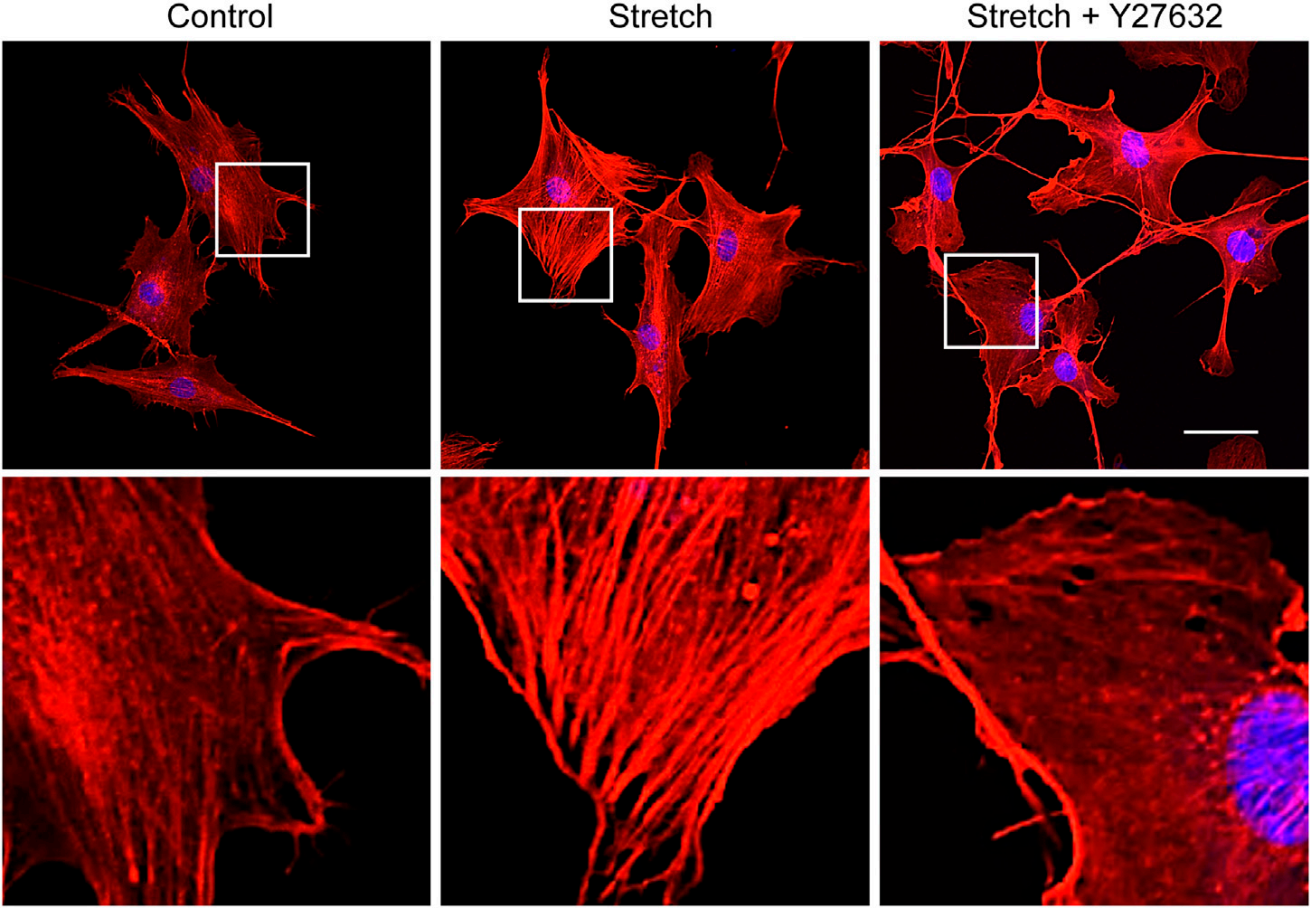
ROCK inhibitor impairs stretch induction of F-actin formation. MSCs were stretched without or with ROCK inhibitor (Y27632) for 1 h, and F-actin was stained with rhodamine phalloidin (red) and nucleus with DAPI (blue). The second row images are magnified views of the white boxes from the first row. Scale bar = 20 μm.

Studies with static mechanophysical cues such as substrate stiffness demonstrated that YAP activity is positively correlated with F-actin construct formation (Totaro et al., 2018). In addition, mechanical stretch loading could facilitate actin cytoskeletal remodeling to assemble F-actin (Thompson et al., 2012). Considering that Rho/ROCK is an upstream signaling regulator of F-actin formation (Andalib et al., 2013), we tested whether Rho/ROCK is involved in regulating the YAP nuclear transport in MSCs under mechanical stretch loading. When MSCs were exposed to cyclic stretch in the presence of Y27632 (a ROCK inhibitor), stretch-induced YAP nuclear import was significantly suppressed (Figures 4A, B), as assessed by immunofluorescent imaging followed by nuclear YAP-positive cell quantification. Consistent with the immunofluorescence, immunoblotting with nuclear and cytoplasmic fractionation also demonstrated that the increase in nuclear YAP residence by cyclic stretch was significantly diminished under Y27632 (Figures 4C, D). We next validated the control of F-actin by ROCK under stretch. Actin stress fiber formation was noticeably strengthened in MSCs exposed to cyclic stretch, while this reinforcement was impaired by Y27632 (Figure 5). These results indicate that cyclic stretch loading induces the development of F-actin via ROCK, which assists the nuclear transport of YAP under stretch loading.

### 3.6 A ROCK blocker, Y27632, inhibits cyclic stretch induction of MSC osteogenesis

**FIGURE 6:**
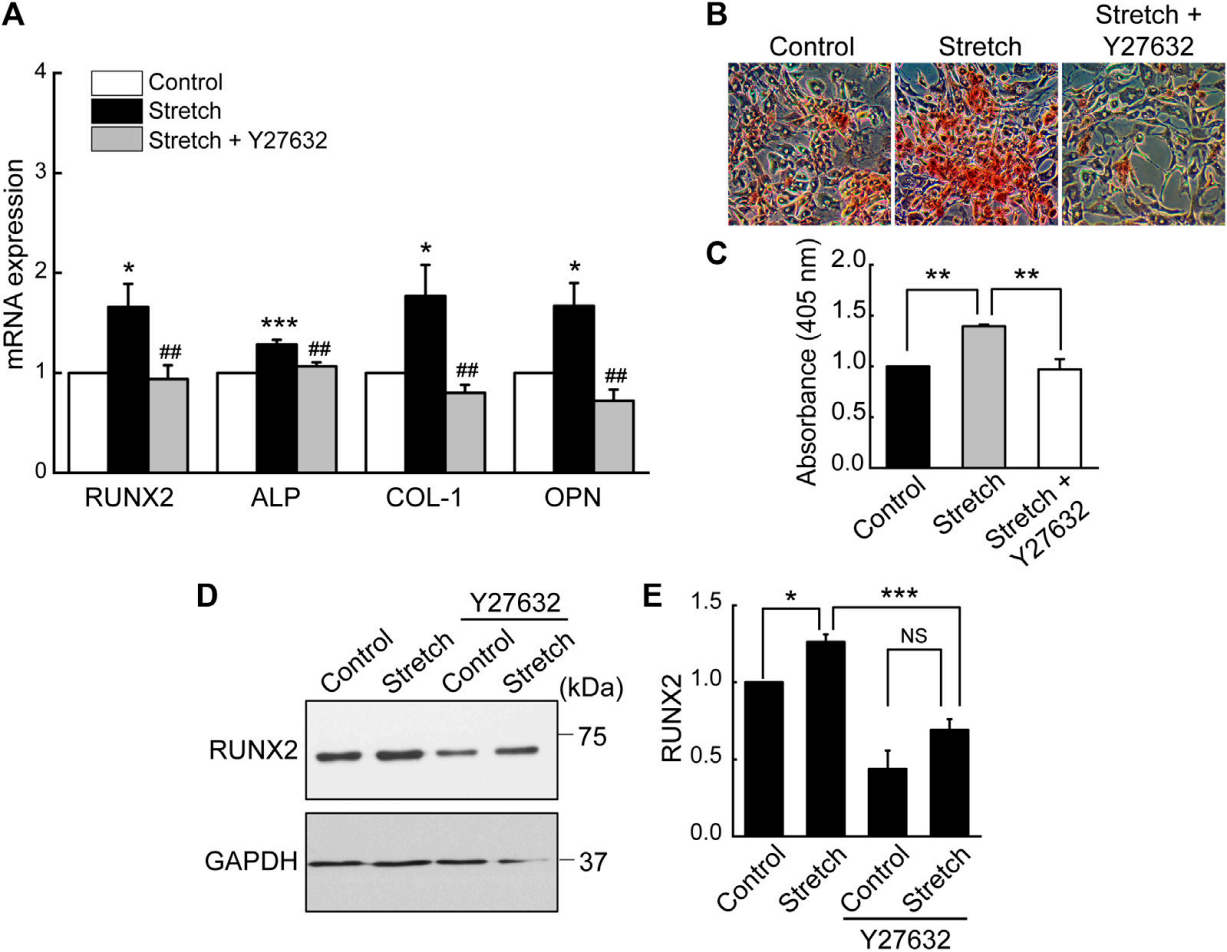
Stretch loading induction of MSC osteogenesis is suppressed by ROCK inhibitor. MSCs were stretched without or with ROCK inhibitor (Y27632). **(A)** Osteogenic gene expressions by qRT-PCR on day 7 (*n* = 4). **(B,C)** Alizarin red bone mineral staining on day 21 (*n* = 3). **(D,E)** Western blotting of RUNX2 expression on day 7 (*n* = 3). NS: non-significant; *: *p* < 0.05, **: *p* < 0.01, and ***: *p* < 0.001 compared with control or corresponding counterpart; ^##^: *p* < 0.01 for comparison between stretch vs. stretch + YAP siRNA.

Based on the result revealing that ROCK is required for stretch induction of YAP nuclear import in MSCs, we examined the effect of ROCK inhibition on MSC osteogenesis under stretch. When cyclic stretch loading was applied under Y27632 (a ROCK inhibitor), stretch-induced increases in MSC osteogenic transcription factor and gene expressions and mineralization were significantly reduced (Figure 6).

## 4 Discussion

We investigated the effects of cyclic mechanical stretch loading on YAP mechanosensing and osteogenic differentiation of MSCs and potential regulatory mechanisms. We observed that cyclic stretch loading triggered nuclear translocation of YAP, a key transcriptional activator, in murine (Figure 1) and human (Supplementary Figure S3) MSCs. YAP nuclear import along with decreased YAP cytoplasmic retention was accomplished via the stretch suppression of YAP phosphorylation at S127 and S397, and the key Hippo signaling pathway (LATS1 phosphorylation) known to affect YAP phosphorylation was also impaired by stretch (Figure 2). Cyclic stretch-induced YAP transcriptional activation via nuclear transport played a significant role in promoting MSC osteogenic transcription and differentiation, as assessed by YAP siRNA (Figure 3). As an underlying mechanism, we showed that YAP activation under stretch was regulated by ROCK, since YAP nuclear import by cyclic stretch loading was significantly suppressed by ROCK inhibitor, Y27632 (Figure 4). In this process of YAP control, ROCK-mediated F-actin formation by cyclic stretch (Figure 5) could play a regulatory role in an analogous manner as suggested for static mechanophysical cues such as stiff substrate culture. Finally, ROCK inhibition abrogated stretch-induced enhancement in MSC osteogenesis (Figure 6), indicating the regulation of a stretch-YAP dynamic mechanosensing-MSC osteogenesis axis via ROCK.

YAP emerged as a central mechanosensor that transduces extracellular mechanophysical stimulatory signals to transcription actions. Activation in YAP via nuclear transport is now proposed as a key determining factor for endothelial cell proliferation and migration (Mascharak et al., 2017), valvular interstitial cell phenotypic change to myofibroblast (Ma et al., 2017), breast cancer cell migration (Chen et al., 2021b), and MSC osteogenic differentiation (Chen et al., 2021a). However, it is still largely unknown how YAP is translocated into the nucleus. While the potent role of YAP as a mechanosensitive transcriptional regulator is recognized as universal, the challenge is that the exact underlying YAP mechanosensing mechanisms may differ with cell types and mechanical stimuli (Dasgupta and McCollum, 2019). Furthermore, the majority of YAP studies for varying types of cells has focused on static mechanophysical cues (matrix stiffness, micropatterned cell size, and substrate topography), including recent reports on MSC osteogenesis (Jiao et al., 2020; Chen et al., 2021a). Here, we show for the first time, to the best of our knowledge, that dynamic mechanical loading, e.g., cyclic stretching, triggers YAP nuclear import in MSCs to activate osteogenic transcription via RUNX2 upregulation thus inducing terminal osteoblastic differentiation.

For MSCs, we showed that cyclic stretch loading at 10% strain and 1 Hz frequency induces YAP nuclear transport (Figure 1), as demonstrated by both immunofluorescent imaging and immunoblotting of nuclear extracts. Our results provide additional evidence along with the study by the Mauck group that showed 3% strain and 1 Hz frequency cyclic stretching induced YAP nuclear transport in MSCs (Driscoll et al., 2015). A recent report from the Rubin’s group showing a contrasting result—no YAP nuclear import in MSCs under cyclic stretching at 2% strain and 10 cycles/min (or 0.167 Hz) (Sen et al., 2022) - suggests that either strain or frequency, or their combination, may affect YAP nuclear transport behavior under cyclic stretch loading. While these studies from Mauck and Rubin groups did not test the role of YAP on MSC osteogenic transcription, our results may suggest an implication for bone tissue engineering and skeletal regenerative medicine. That is, mechanical stretch loading regimens need to be better adjusted to induce YAP nuclear import considering its potent role in triggering RUNX2 osteogenic transcription and terminal osteogenic differentiation (Figure 3). One can refer to a recent study on fibroblasts that reported systematic and thorough data on the combinatory control of YAP nuclear import by cyclic stretching at varying strains (2.5%–20%) and frequencies (0.125 Hz–2 Hz) (Andreu et al., 2021).

Knowing that cyclic stretching at a specific regimen induces YAP nuclear import in MSCs, one of the pivotal tasks is to examine if anti-YAP Hippo pathway is involved. It is known that Hippo acts to oppose YAP activity through the phosphorylation of LATS1/2 with accessory proteins such as MOB. Phosphorylated LATS1/2 induces YAP phosphorylation, thereby sequestering YAP within the cytoplasm to prevent its nuclear entry. Static culture studies proposed that YAP activation could be achieved via Hippo or not, depending on cell types and mechanophysical cues (Dasgupta and McCollum, 2019). In our study on the cyclic stretch loading of MSCs, we demonstrated that cyclic stretch loading significantly suppressed YAP phosphorylation at two major phosphorylation sites, S127 and S397, as well as Hippo signaling (p-LATS1) (Figure 2). These data point to a speculation that Hippo could be involved in the cyclic stretch loading induction of YAP nuclear import in MSCs. A further study repeating the test with LATS inhibition/activation will reveal the Hippo-YAP pathway in MSCs under stretch. Intriguingly, one recent study testing the fluid shear stimulation of endothelial cells proposed that flow shear stress triggers YAP nuclear import in endothelial cells in a Hippo-independent way, since LATS1/2 phosphorylation did not change with fluid shear (Nakajima et al., 2017). The comparison of these results, MSC cyclic stretching vs. endothelial cell flow shearing, supports that the dynamic mechanical loading induction of YAP nuclear transport may or may not be associated with Hippo signaling, potentially altering with cell types and mechanical stimulation modes.

The underlying mechanisms of cells sensing extracellular mechanophysical cues and transducing them to adjust YAP activity are not fully understood yet. Studies on static mechanophysical cues have proposed a regulatory role of cytoskeletal architecture and tension in controlling YAP. A pioneering study demonstrated that YAP nuclear localization was increased on substrates that promoted stressed F-actin formation, e.g., a stiff substrate or large micropatterning cell size relative to soft substrate or small micropattern (Dupont et al., 2011). Based on this, a comparative role of RhoA-ROCK (an upstream of F-actin) and myosin II (a player to generate F-actin contractility) in YAP activation was tested. It was proposed that the disruption of F-actin integrity may be more potent to prohibit YAP nuclear import than inhibiting myosin contractility by testing Y27632 (ROCK inhibitor) vs. blebbistatin (non-muscle myosin II blocker) on substrates with varying stiffness (Das et al., 2016). In one mechanical stretch loading study with periodontal ligament cells, YAP nuclear transport by stretch was suppressed by both ROCK inhibitor and non-muscle myosin II blocker (Yang et al., 2018). For MSCs, we demonstrated that ROCK inhibitor (Y27632) significantly impaired the cyclic stretch loading induction of YAP nuclear transport (Figure 4) and F-actin formation (Figure 5), suggesting a regulatory role of the upstream effector of F-actin, RhoA-ROCK, in controlling YAP. ROCK inhibition further suppressed stretch enhancement of MSC osteogenesis (Figure 6). Our results suggest that cyclic stretch loading of MSCs may share the YAP mechanosensing mechanism with the stiff substrate culture such that improved F-actin formation via RhoA-ROCK is the element to direct YAP activation via nuclear localization.

A complexity in the YAP mechanosensing mechanism is that mechanosensing elements other than F-actin, such as focal adhesion on which F-actin is anchored, can also affect the YAP action. For static mechanophysical cues, recognizing stiff substrate induction of YAP activation, integrin-mediated focal adhesion reinforced by stiff substrate culture was proposed as another YAP-regulatory component. Among focal adhesion mechanosensors (integrin, talin, paxillin, vinculin, and focal adhesion kinase or FAK), FAK has been proposed to play a key role for YAP mechanosensing under static culture (Lachowski et al., 2018). In a recent mechanical stretch loading study with fibroblasts, inhibiting integrin αVβ3-based focal adhesion impaired the stretch activation of FAK and YAP (Peng et al., 2021). However, there is little evidence for the direct FAK control of YAP nuclear transport under stretch. Since it is generally understood that focal adhesion and F-actin mutually share mechanophysical signals via outside-in and inside-out signaling (Shen et al., 2012; Murphy and Brinkworth, 2021), both may play an associated role in YAP mechanosensing. However, currently, little is known on the collaborative impact of focal adhesion and F-actin in YAP nuclear import under dynamic mechanical loading. Upcoming studies need to tackle the collaborative role of focal adhesion and F-actin, and even with the other side of the F-actin linkage - the linker of nucleoskeleton and cytoskeleton (LINC)—in regulating YAP mechanosensing under dynamic mechanical loading.

It is notable that dynamic mechanical loading cues other than stretching have also been tested for the MSC osteogenesis via YAP/TAZ. These include fluid shear stress stimulations (Zhong et al., 2013; Kim et al., 2014) and even acoustic tweezing (Xue et al., 2017). The study by Zhong et al. (2013) showed that flow shear-activated YAP in MSCs induced an increase in osteogenesis and a decrease in adipogenesis, similar to our result from cyclic stretching (Supplementary Figure S2). Together, these provide evidences for dynamic mechanical loading to direct MSC fate decision in osteogenesis vs. adipogenesis via YAP nuclear import. This thereby suggests a mechanism for previously reported mechanical induction of MSC osteogenesis at the expense of adipogenesis (David et al., 2007). With respect to the underlying mechanistic pathway, those flow shear studies also proposed the role of F-actin in YAP activation by using Y27632 (ROCK inhibitor) and cytochalasin D (F-actin inhibitor). More studies on the comparative effects of mechanical stretch loading and flow shear stress on YAP nuclear import for osteogenic transcription at various mechanical loading regimens (e.g., strain, shear stress, etc.) may provide information on the loading cues optimal for improved bone tissue formation with MSCs.

In conclusion, this study examined the YAP mechanosensing in MSCs under cyclic mechanical stretch loading and its impact on MSC osteogenic transcription and differentiation. We showed that cyclic stretching at 10% strain and 1 Hz frequency triggered YAP nuclear transport in MSCs. This stretch-induced YAP nuclear import was achieved by the decrease in YAP phosphorylation at S127 and S397. Moreover, the anti-YAP-regulatory Hippo pathway, LATS1 phosphorylation, was suppressed by cyclic stretch. The cyclic stretch induction of MSC osteogenic transcription and differentiation was mediated by YAP nuclear import, as YAP siRNA impaired the stretch-induced MSC osteogenesis. We also showed that YAP nuclear transport in response to cyclic stretch was abrogated by ROCK inhibitor, suggesting the involvement of ROCK and the associated downstream cascade such as F-actin in the dynamic mechanical loading activation of YAP. Accordingly, stretch induction of MSC osteogenesis was suppressed by inhibiting ROCK. Our data may provide an implication in bone tissue engineering and skeletal regenerative medicine that utilize MSCs. Specifically, dynamic mechanical loading control of YAP-mediated osteogenic transcription in MSCs may provide insights into both underlying mechanotransduction mechanisms and required mechanical loading tools.

## Data Availability

The original contributions presented in the study are included in the article/Supplementary Material, further inquiries can be directed to the corresponding author.
