# Supplementary material for: YAP mechanotransduction under cyclic mechanical stretch loading for mesenchymal stem cell osteogenesis is regulated by ROCK
Source: Front Bioeng Biotechnol. 2024 Jan 11;11:1306002. doi: 10.3389/fbioe.2023.1306002 (PMC10809151; doi:10.3389/fbioe.2023.1306002)
Supplement: Supplementary file 1 [file Presentation1.pdf]

YAP mechanotransduction under cyclic mechanical stretch loading for mesenchymal stem cell osteogenesis is regulated by ROCK

Eunju Kim, Brandon D. Riehl, Tasneem Bouzid, Ruiguo Yang, Bin Duan, Henry J. Donahue, Jung Yul Lim

---

Supplementary Material

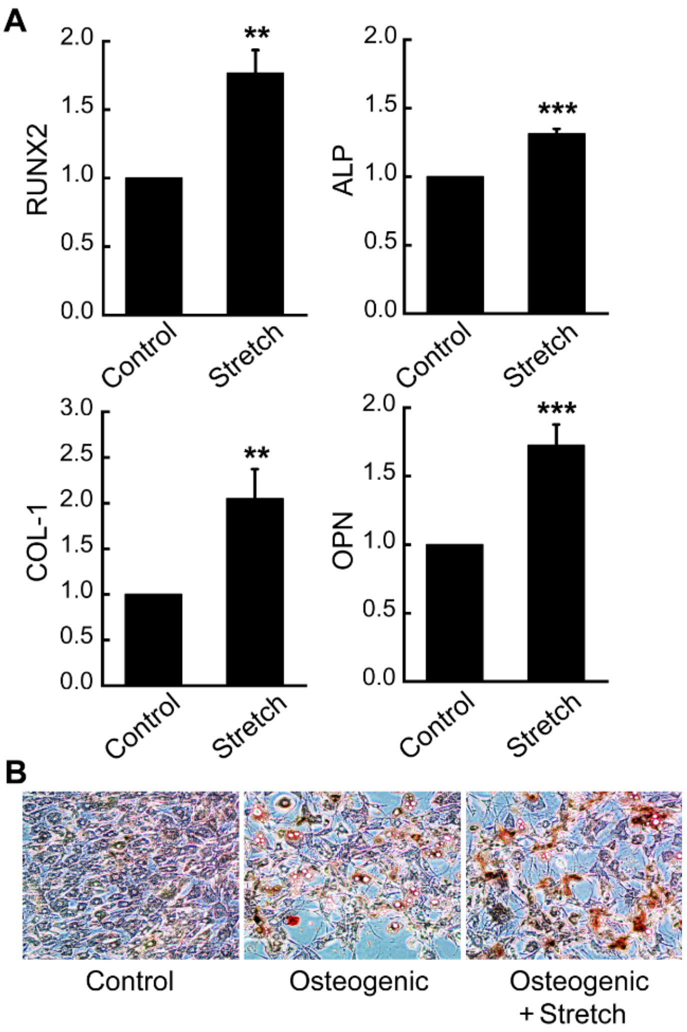

Supplementary Fig. S1. Cyclic stretch promotes the osteogenic differentiation of MSCs. MSCs were stretched for 3 days (1 h/day) with the onset of the exposure to osteogenic induction media. (A) Osteogenic gene expressions by qRT-PCR on day 7 (n = 6). (B) Alizarin red bone mineral staining on day 21. **: $p < 0.01$ and ***: $p < 0.001$ compared with control.

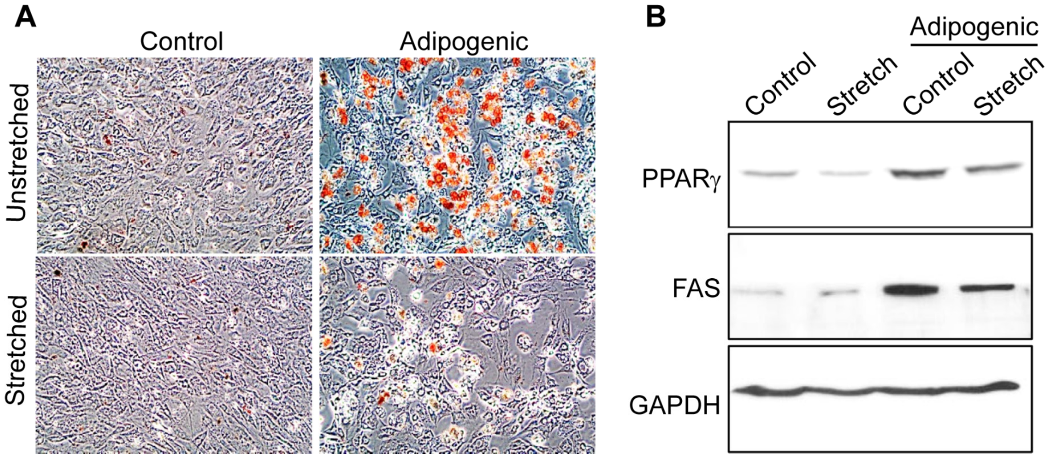

Supplementary Fig. S2. Cyclic stretch suppresses the adipogenic differentiation of MSCs. MSCs were seeded on the stretch plate for 2 days until they reached confluence. Adipogenesis was induced by exposure to 1 μg/ml insulin, 0.5 mM isobutyl-methylxanthine, and 10 μM dexamethasone for 2 days, and stretch was applied for 1 h/day. After that, adipogenic maintenance medium (1 μg/ml insulin) was given for additional 8 days. (A) Lipid accumulation shown by oil red O staining. (B) Western immunoblotting of adipogenic markers, PPARγ and FAS, after 2 days of adipogenic induction without or with stretch.

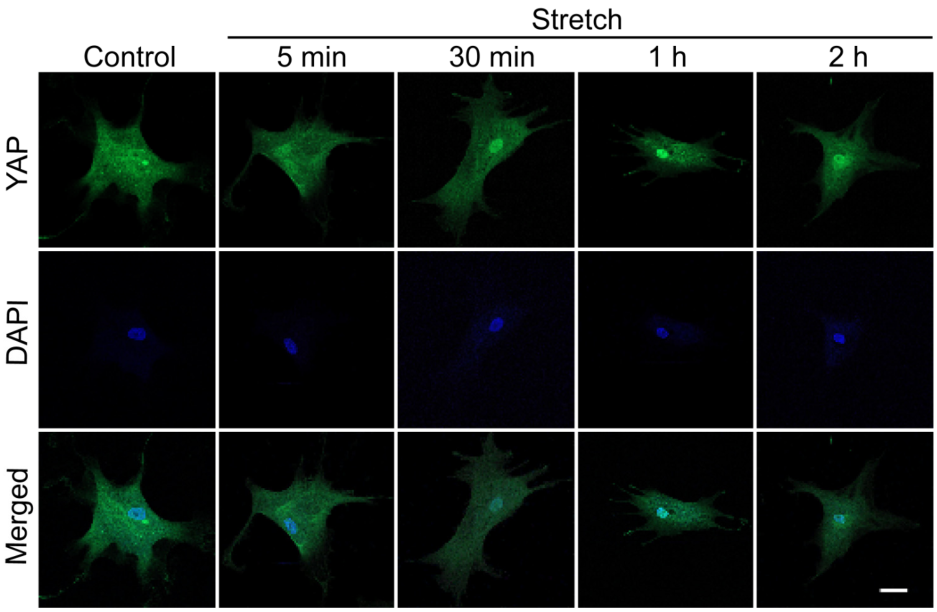

Supplementary Fig. S3. Human MSCs show cyclic stretch-induced YAP nuclear transport. hMSCs (Lonza, PT-2501, 25 year-old female) were exposed to cyclic stretch loading at 10% strain and 1 Hz frequency. Immunofluorescent imaging of YAP (green) and DAPI (blue). Scale bar = 20 μm.

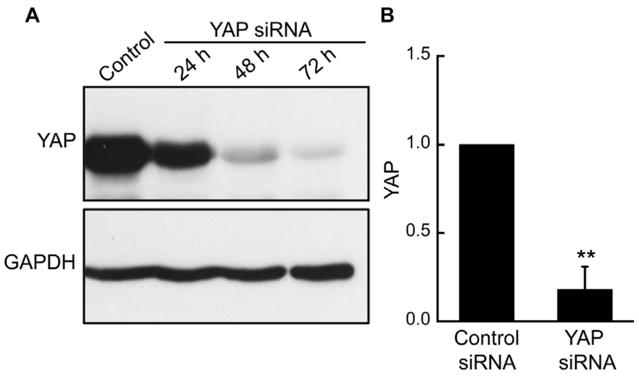

Supplementary Fig. S4. YAP silencing via siRNA transfection. (A) MSCs were transfected with control siRNA or YAP siRNA, and YAP expression was assessed by western blotting. (B) At 48 h post-transfection, YAP expression was significantly decreased (n = 3). **: $p < 0.01$.
